# Evaluation of the costs of agricultural diffuse water pollution abatement in the context of Lithuania’s water protection goals and climate change

**DOI:** 10.1007/s00267-022-01745-1

**Published:** 2022-11-11

**Authors:** Svajunas Plunge, Mindaugas Gudas, Arvydas Povilaitis, Mikołaj Piniewski

**Affiliations:** 1 Department of Hydrology, Meteorology and Water Resources, Warsaw University of Life Sciences, Nowoursynowska st. 159, Warsaw, 02-776 Poland; 2 Institute of Water Resources Engineering, Vytautas Magnus University, Universiteto st. 10, Kaunas district, Akademija, LT-53361 Lithuania; 3 Hydrographical Network Division, Environmental Protection Agency, Juozapaviciaus st. 9, Vilnius, LT-09311 Lithuania

**Keywords:** Best management practices, Pollution abatement, SWAT model, Genetic algorithm, Lithuania

## Abstract

This study aimed at evaluating the scale and costs of an environmentally and economically optimal set of Best Management Practices (BMPs) for agricultural pollution abatement in Lithuania in order to reach water protection goals in both inland and marine waters by distributing BMPs optimally in space, while taking climate change impacts into consideration. The assessment of BMPs impact involved the use of the SWAT model by applying two climate change representative concentration pathways (RCP4.5 and RCP8.5) and two time horizons (mid-century and end-century), as well as five BMPs (arable land conversion to grasslands, reduced fertilization, no-till farming, catch-crops, and stubble fields throughout winter). The optimization of the set of BMPs employed a genetic algorithm. The results suggest that the need for BMPs application will increase from 52% of agricultural areas in the historical period up to 65% by the end of century in the RCP8.5 scenario. This means less arable land could actually be used for crop production in the future if water protection targets are met. The high costs for reaching water targets would rise even more, i.e. by 173% for RCP4.5, and by 220% for the RCP8.5 scenario, reaching approximately 200 million euros/year. In such a context, the BMP optimization approach is essential for significant reduction of the costs. Winter cover crops and reduced fertilization show the best effectiveness and cost balance, and will therefore be essential in pursuing water protection targets.

## Introduction

Around 50% of surface water and 25% of groundwater bodies in the EU were not in good status in 2016 in accordance to the requirements of the Water Framework Directive (WFD), and agricultural activities are among the main reasons for the deterioration of water bodies according to the European Environmental Agency EEA (2020). The majority of European policy instruments for water protection regulation emphasize the importance of targets and regulation for total nitrogen (TN) and total phosphorus (TP) (Grizzetti et al. 2021). TN and TP are nutrients crucial in agricultural production, but also key substances driving eutrophication processes in water bodies. Eutrophication leads to the deterioration of ecosystems, summer cyanobacterial blooms, and extensive bottom water hypoxia in surface waters, with increasing frequency and scale in the Baltic Sea (Carstensen et al. 2014).

The Baltic Sea is suffering from eutrophication, affecting 97% of its territory (HELCOM 2018a). Around 47% of TN and 36% of TP riverine loads to the Baltic Sea was generated by diffuse sources mainly from agriculture (HELCOM 2018b). These sources are among the most significant pressures on surface water bodies, severely affecting around 38% of EU water bodies (EEA 2018), as well as 42% of water bodies in Lithuania (Aplinkos apsaugos agentūra 2021a). Abating this source of pollution is therefore a precondition for protecting water bodies and water ecosystems. Yet, the implementation of agricultural diffuse pollution control policies has been ineffective due to reasons, such as improper design of policies, lack of the targeted approach, low priorities, or no integration with farming economic models (Brady et al. 2021; Thorsøe et al. 2021). According to Andersson et al. (2021), cost-effectiveness thinking is missing from the design of water pollution abatement policies, as is the simultaneous consideration of several environmental objectives.

Climate Change (CC) is widely anticipated to change water flows, making dry areas drier, and wet areas wetter (Lobanova et al., 2018; Marx et al. 2017; Schneider et al. 2013). Moreover, many authors (Barclay and Walter 2015; Øygarden et al.; 2014) expect increased nutrient losses (primarily nitrogen) from soils. In the Nordic-Baltic region, higher nitrogen mineralisation due to rising temperatures and precipitation in cold periods are expected to increase leaching of nitrates (Molina-Navarro et al. 2018). Higher nutrient loads together with the likely prolongation of the growing period could further exacerbate the already severe eutrophication problems in places such as the Baltic Sea (HELCOM 2018a).

Therefore, multiple authors advocate (Brouziyne et al. 2018; Qiu et al., 2020; Renkenberger et al. 2017; Wallace et al. 2017; Xu et al. 2019) for accounting for changes expected due to CC when designing the strategy for effective agricultural diffuse water pollution reduction. Various studies show (Bosch et al. 2018; Qiu et al. 2020; Renkenberger et al. 2017) that the costs of meeting water quality goals are likely to increase due to CC impacts as Best Management Practices (BMPs) will become less effective or/and diffuse water pollution will increase. Moreover, changes in spatial distribution patterns of water pollution are also expected (Renkenberger et al. 2017). This is important, because targeting BMPs in Critical Source Areas (CSAs) yields far better results for pollution reduction (Piniewski et al., 2021; Wagena and Easton 2018; Xu et al. 2019). Beside water pollution reduction, agriculture should be maintained economically viable in future climate, which is already an enormous challenge considering the expected CC impacts. Consequently, the protection of aquatic systems should be coupled with the strengthening of the resilience of agricultural systems (Bosch et al. 2018; Brouziyne et al. 2018; EEA 2020).

Since testing of different pollution abatement strategies is too costly in real life, a large number of hydrological or ecohydrological models has been created and used extensively for this purpose (Andersen et al. 2006; Barclay and Walter 2015; Hesse et al. 2014; Huttunen et al., 2021,2015; Olsson et al. 2016; Vansteenkiste et al., 2013). The Soil and Water Assessment Tool (SWAT) model is one of the most extensively used among them (Gassman and Yingkuan 2015). This semi-distributed process-based ecohydrological model is used to simulate quality and quantity of surface and groundwater (Arnold et al. 1998), and to answer environmental impact, water management, or hydro-climatic questions (Tan et al. 2020). Multiple studies have employed it for questions related to the impact of CC on agricultural pollution (Bosch et al. 2018; Brouziyne et al. 2018; Jeon et al., 2018; Qiu et al. 2020; Teshager et al. 2017; Wagena and Easton 2018; Woznicki and Nejadhashemi 2014). SWAT has been extensively applied for investigating water policy and management questions (Molina-Navarro et al., 2018; Piniewski et al. 2021; Thodsen et al. 2017). Furthermore, a number of studies integrated the model with Genetic Algorithms (GA) for delivering optimized solutions for BMPs selection and spatial distribution for reaching water protection goals (Geng et al. 2019; Kaini et al. 2012; Naseri et al., 2021).

The year 2021 was an important milestone for the countries of the Baltic Sea Region (BSR) with regard to water protection. The Baltic Sea Action Plan (BSAP) to reach good environmental status in the marine environment of the Baltic Sea was updated by the Helsinki Commission (HELCOM). Moreover, the third cycle River Basin Management Plans (RBMP) were drafted by national authorities of the EU Member States with a view to implement the WFD. These are two crucial and connected pillars for water protection in the BSR, since both depend on the efforts made in river basins However, scarce attempts have been made to harmonize the targets set by these important documents, particularly in the east of the region. Even less information is available on the scale needed to reach integrated water protection goals, especially taking into account CC impacts and the scale of the entire country. Finally, information about the impact of CC on potential costs of meeting water targets is relatively rare. Yet, it is becoming more and more crucial for proper long-term planning, CC adaptation, as well as environmental target setting.

The objectives of this study are as follows: (1) Link marine and inland surface water protection goals for Lithuania’s case; (2) Evaluate the scale and costs of an environmentally (taking into account only water protection) and economically optimal set of BMPs for agricultural pollution abatement in order to reach water goals; (3) Elaborate on the optimal spatial distribution of BMPs permitting reaching marine and inland surface water goals with the lowest costs; (4) Assess how the scale and cost of a possible optimal agricultural pollution abatement program required to reach water protection goals will be affected by CC.

## Materials and methods

The study is based on the River Modelling System (RMS) of the Environmental Protection Agency of Lithuania (EPA), developed by integrating the SWAT model. The description of the system, the used data, preparation steps, calibration, and validation for hydrology and water quality are available online^1^ in the report produced by PAIC (2015). The results of the application of RMS for the assessment of CC impact on water bodies in Lithuania is presented in the article by Plunge et al. (2022a). The results of the utilization of BMPs in RMS in the context of CC impacts is presented in the article by Plunge et al. (2022b). This paper is a continuation of a previous work already published by Plunge et al. (2022a) and Plunge et al. (2022b). This study therefore only provides information needed to understand the applied methods, directly relevant to the study, without going into details already presented in the aforementioned studies.

### Study area

The entire territory of Lithuania was selected as the study area, covering all inland surface water bodies. The selection was intentional for addressing large-scale water management issues and goals set at the national level. Small-scale studies are essential in detailed discussions, where precise local understanding and locally-tailored solutions are needed. Yet the extrapolation of conclusions to a country scale always involves an additional level of simplification and risk of overlooking important parts of the system that can be avoided by preparing modelling tools for the entire country or its region (if a country is relatively large).

**Fig. 1:**
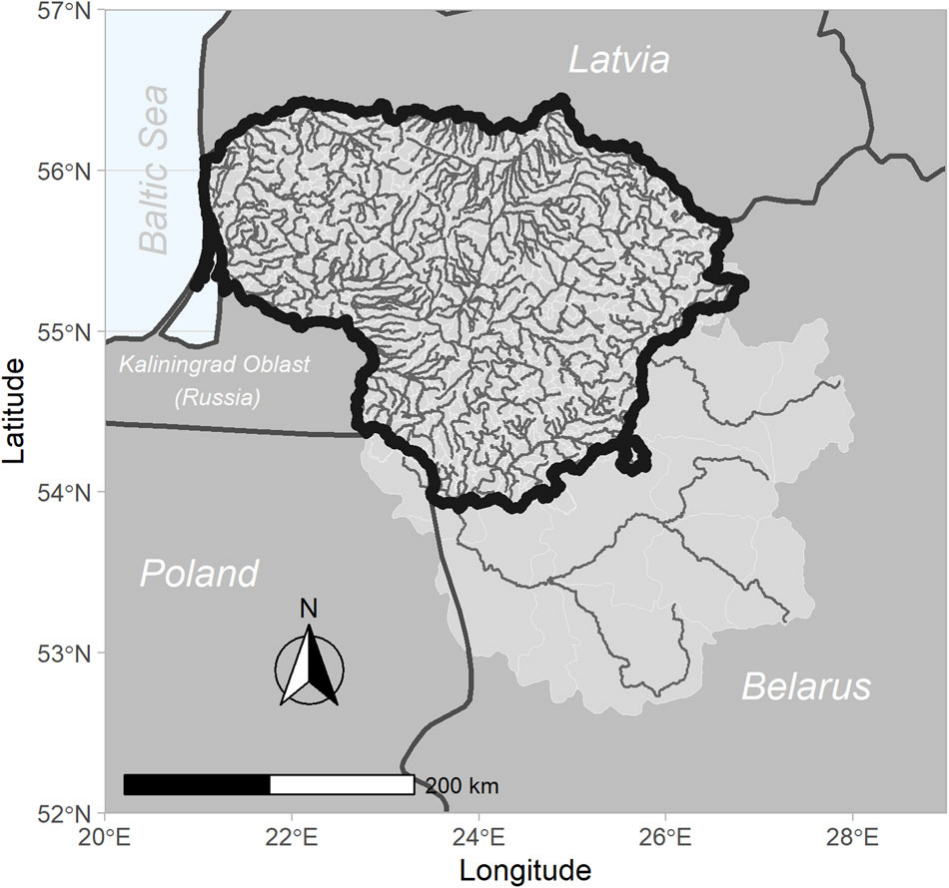
Area covered by the RMS (bold line marks the territory of Lithuania, light grey – area covered by the model, small grey lines – water bodies represented in the model)

The whole country model used in the study also includes transboundary river basins in the neighboring countries that provide inflow into the rivers of Lithuania. The area covered by RMS is presented in Fig. 1 (Plunge et al. 2022a). It encompasses the entirety of Lithuania with an area of 65,300 km^2^, a part of Belarus – 45,951 km^2^, and a part of Poland – 2516 km^2^. Results presented in the article, however, cover only the territory of Lithuania. The dominant land use type here is agriculture, occupying 59% of Lithuania, followed by forests – 30%, water bodies – 2%, artificial areas (urban, industrial, transport, mine, dump and construction sites) – 3%, and other areas – 6% (PLC-7 2019). While addressing pollution impacts on inland waters, the RMS also covers interactions with the marine environment of the Baltic Sea as it provides information about nutrient loads into the Sea according to selected scenarios, which can be assessed in terms of load comparison against specific marine-related load targets.

### Climate change data

The European Coordinated Regional Climate Downscaling (EURO-CORDEX) program^2^ data of a resolution of 0.11 degrees or ~12.5 km (EUR-11) were used for the study. Seven different Regional Climate Models (RCM) with the boundary conditions of several Global Climate Models (GCM) were selected. The selection of multiple datasets is recommended by many authors (Rathjens et al. 2016; Singh 2016) to encompass the variation connected to CC data. The selection of CC RCM models used in this study and the related data summary are presented in Table 1 (first presented in the article by Plunge et al. (2022b)).

**Table 1:** RCMs and GCMs model combinations selected for the study, and their mean deviations from the historical period (M – mid-century, E – end-century)

Variable	Precipitation mm/d				Min. daily temperature °C				Max. daily temperature °C			
Representative Concentration Pathway	RCP 4.5		RCP 8.5		RCP4.5		RCP8.5		RCP4.5		RCP8.5	
RCM & GCM Combination \ Period	M	E	M	E	M	E	M	E	M	E	M	E
CCLM4-8-17 (CLMcom) MPI-ESM-LR (MPI)	0.1	0.1	0.1	0.4	0.7	1.4	1.3	3.4	0.5	1.2	1	2.8
HIRHAM5 (DMI) HadGEM2-ES (MOHC)	−0.1	0.1	0.1	0.2	3	3.9	2.8	7.2	3.4	4.4	3.3	8.4
RACMO22E (KNMI) EC-EARTH (ICHEC)	0.1	0.1	0	0.1	1.3	3	1.5	5.6	1.6	3.3	1.7	6
RCA4 (SMHI) NorESM1-M (NCC)	0.1	0.2	0.2	0.4	2.6	3.5	2.7	6	2.9	4	3	6.9
REMO2009 (MPI-CSC) MPI-ESM-LR (MPI)	0.2	0.1	0.2	0.4	0.7	1.3	1.1	3.2	0.4	1.1	0.9	2.3
REMO2015 (GERICS) NorESM1-M (NCC)	0.1	0.1	0	0.1	1.8	2.5	1.6	4.2	1.6	2.3	1.5	3.9
WRF381P (IPSL) IPSL-CM5A-MR (IPSL)	0.2	0.3	0.6	1	1.1	2.2	1.9	4.3	0.8	1.8	1.4	3.4

Only daily precipitation and minimum and maximum temperature data were used from the RCMs dataset. The remaining required weather data were prepared using the internal SWAT module of the weather generator. Statistical bias-correction methods of Local Intensity Scaling and Linear Scaling were applied for temperature, and Distribution Mapping for precipitation with the aid of CMHyd program (Rathjens et al. 2016). Data from 18 national stations were used. Two emission scenarios were selected for the study. The Representative Concentration Pathway (RCP) scenario RCP4.5 was selected to represent the “intermediate” case situation in which global temperature warming would be averaging approximately 1.8 °C by the end of the century. The RCP8.5 scenario was selected to represent the “worst-case” situation where an average warming of around 3.7 °C (IPCC 2014) for the same time horizon is expected. Selected scenarios allowed for examining the likely range of future conditions, while using computational resources efficiently. Three periods of 20 years were selected for the assessment and presentation of results. The historical period included the interval 2000–2019, mid-century – the period 2040–2059, and end-century – 2080–2099. More details are provided in the article by Plunge et al. (2022a).

### Best management practices

Five BMPs effective for agricultural diffuse water pollution abatement were selected. These measures were chosen on the basis of their diffuse water pollution reduction potential, feasibility of costs, consideration in the national River basin management plans (Aplinkos apsaugos agentūra, 2021a) and the possibility to model with SWAT. More information on the design of measures for modelling, their selection, and effectiveness in the context of CC impacts are presented in the article by Plunge et al. (2022b). The following BMPs were selected for the study:

1. Converting arable land to perennial grasslands – *Grassland*+;
2. Reduced fertilization (by −10% from baseline level) – *Fertilization-*;
3. Applying no-plough or no-till farming technology – *No-plough*;
4. Planting cover crops (catch crops) and keeping them through winter – *WintCrops*;
5. Leaving stubble fields through winter – *StubbleFields*.

The selected BMPs were used to run the SWAT model with parameters presented in the report of PAIC (2015). The grassland BMP was applicable (could be modelled due to land use or/and soil type related constraints) to a maximum of 21.4% of the country’s territory, reduced fertilization – 37%, no-plough technology – 14.3%, winter cover crops – 13.9%, and stubble fields through winter – 13.4%. The scenario with no BMPs applied was called *no BMP*. Notice that the areas presented above overlap to a certain extent and that BMPs might be combined in certain cases (i.e. fertilization reduction could be used with winter cover crops, etc.). However, the subsequent optimization procedure did not include the assessment of combined measures or the application of them in a sequential order on the same area.

### Optimized selection of BMPs

Results presented in Plunge et al. (2022a) and Plunge et al. (2022b) were used as input for this study in order to determine the required scale (area of BMPs application), composition (proportions necessary for BMP areas), spatial distribution, and costs of measures to reach the BSAP and WFD goals for Lithuania, and to determine their shift with consideration of CC impact.

#### Targets

Updated country-basin nutrient input ceilings (NIC) for BSR countries are presented in the HELCOM (2021) report in Tables 6 (for TN) and 7 (for TP). NIC represents the maximum yearly net load contribution to the Baltic Sea, which countries are allowed to release. Lithuania’s rivers contribute loads only to the Baltic Proper (BAP) and the Gulf of Riga (GUR) Baltic Sea basins, dividing the country into parts of 77 and 23% (Northern part), respectively. For BAP, Lithuania’s maximum allowable annual loads are 25878 t/y for TN and 703 t/y for TP. In the GUR basin, Lithuania is restricted to an annual NIC of 8820 t/y TN and 175 t/y TP.

The methodology for calculating national loads of TN and TP is described in the HELCOM pollutant loads compilation guidelines (HELCOM 2019) and detailed in the EPA technical paper (Aplinkos apsaugos agentūra 2014). Those documents describe how pollution loads should be calculated from concentrations and water flows, how data gaps should be filled, and how transboundary loads should be eliminated. Moreover, the documents specify which specific monitoring points should be used for the Lithuanian load calculation, how loads should be calculated for unmonitored areas, how special cases on border areas should be dealt with, how loads should be normalized to eliminate impact of water flow variation, and many other details.

These methodologies were used in the calculation of concentrations for water bodies, in line with the achievement of NIC for Lithuania. Only the riverine pathway was assessed, ignoring loads reaching the Baltic Sea via atmospheric pathway (atmospheric deposition) and direct point sources. Long term (1995–2020) contributions of those sources from Lithuania are around 7 and 0.9% of the country ‘s riverine nitrogen loads respectively. To obtain maximum concentrations in water bodies that would satisfy HELCOM targets, nutrient loads to BAP and GUR basins were calculated iteratively, starting with very low concentrations (assuming all water bodies have the same concentration^3^), and raising them by a marginal value in each loop. Then, with each new TN and TP concentration, Lithuania’s loads to both Baltic Sea basins were recalculated and checked against NICs. The last concentrations that would allow avoiding exceeding NIC targets by nutrient loads from Lithuania to the Baltic Sea were saved. It was assumed that if these concentrations were set as maximum allowable concentrations (MAC) for all water bodies, it would be sufficient to keep nutrient loads under NIC (or reach the Baltic Sea protection targets). Water discharge values required for nutrient load calculations were obtained from modelling, and averaged for the evaluation period used in the study.

Lastly, concentrations necessary for BSAP implementation were compared against WFD water body “good status” concentrations (AM 2007) which in the case of Lithuania were 3 mgN/l for TN and 0.14 mgP/l for TP for river water bodies (only rivers export loads directly to the Baltic Sea or other countries). More strict concentrations (NIC or WFD) were applied for setting MAC concentration to be reached in the optimization. This allowed for the assessment of a scenario where both BSAP and WFD targets are reached simultaneously.

#### Optimization

The process of selecting measures for each water body commenced by calculating the required TN and TP load reduction to reach MAC concentration in the no BMP scenario for all CC data sets and model sub-basins (Lithuania model was composed of 1237 model sub-basins). Then each BMP scenario was modelled separately for all CC datasets. Measures were applied only to the applicable Hydrologic Response Units (HRUs)^4^. Sub-basin and HRU model outputs were saved and used further in the assessment. A total of 84 model runs were executed (7 CC datasets × 2 RCP scenarios × 5 BMP scenarios + 7 CC × 2 RCP scenarios for no BMP scenario). Their results were used in the optimization.

HRU level BMP impacts on TN, TP loading to water bodies, and crop yield changes were calculated for each time period. The same CC and RCP datasets were used in calculating BMP impacts between the no BMP and BMP scenario. These HRU level results were further used in the genetic algorithm (GA) constructed for the optimization of measures. GA was used to optimize TN and TP load reduction, pollution abatement costs, and loss of crop yield. The task of optimization here was to select the most optimal measure for each HRU with a view to achieve the desired MAC in each water body in the most optimal way (in terms of effects on concentrations, pollution load reduction, total abatement costs, and loss of crop yield).

#### Genetic algorithm (GA)

The study involved the construction of a simple GA. The GA is based on the foundations of evolutionary biology, and features several characteristic operations, including initial population building, fitness evaluation, sorting, selection, crossover, and randomized mutation. Each of them has specific parameters that control operations. The primary characteristic of GA, however, is the translation of the optimization problem into genetic terms, and using evolutionary mechanics to find sub-optimal solutions where finding the optimal solution is not possible (or requires excessive resources). The study lends itself to the application of such an approach, since the number of solutions could potentially be as high as 6^31613^ = 4.95996233 × 10^24599^ taking into account 6 outcomes for each HRU (no BMP and any one of 5 BMPs) and 31613 (out of a total HRU number of 59839) agricultural HRUs available in the model. This number should be reduced to some extent, because some agricultural HRUs are not suitable for some BMPs, and some sub-basins in the model require no reductions. Nevertheless, it is still not possible to examine such a number of complex solutions.

Each sub-basin was therefore transcribed into a chromosome (representing one individual in a population), with a number of genes corresponding to the number of HRUs in this sub-basin. Each gene had a maximum of 6 distinct alternatives – no BMP and 5 BMPs (the combinations of different measures were considered in the study). Then, a random population of individuals was built for each sub-basin where reductions were required (one individual – one set of all sub-basin’s HRUs, eligible for the application of BMPs, with appropriate selected BMP alternatives; second individual – another set of the same HRUs representing a different BMP combination, etc.). The fitness of each individual was then evaluated.

The evaluation was based on values of four criteria. Two of them were TN and TP load reductions. Third was the cost of abatement. Cost values of each BMP are obtained from the report of PAIC (2015) Table 112. Valuations represented installation, maintenance and income loss compensation expenses per area unit (ha) of measure per year. Full environmental and social aspects (represented by cost-benefit assessment) were not considered as valuation of it was not available for us. The last criteria was a yield loss. Based on these criteria a rank number was calculated and compared to others in a population. Each rank evaluation value consists of the sum of rank values pertaining to each factor (TN, TP load reduction, yield loss, and abatement costs), adjusted by special weights for each factor. Therefore, the fitness value was calculated using Eq. (1).

1 $$\begin{array}{l}Fitness = rank_{TN} \times weight_{TN} + rank_{TP} \times weight_{TP} \\\qquad\qquad+\, rank_{Yield} \times weight_{Yield}+ rank_{Cost} \times weight_{Cost}\end{array}$$

Equation (1). Fitness calculation equation.

For example in GA, 100 individuals were used for optimization. The one with the highest reduction of TN would get 100 value, and that with the smallest – 1. This value would then be modified with weight used for TN reduction. In a similar fashion, values would be assigned to the reduction of TP, cost lowering, and yield preservation. Then, all these values were added together for a final score (rank), and based on those final fitness values, individual solutions were ranked. The best solutions were retained in building the next generation.

The main settings used in GA optimization were determined by examining results from several test runs. Initial weights were based on the ranking of factors for their assumed importance in the agricultural diffuse pollution abatement program. Settings for GA optimization were as follows:

- Population size – 100;
- Selection ratio – 0.1;
- Mutation probability ratio – 0.01;
- Maximum number of generations – 100;
- Initial weights *weight*_*TN*_ = 0.4, *weight*_*TP*_ = 0.2, *weight*_*Yield*_ = 0.1, *weight*_*Cost*_ = 0.3;
- Maximum weight – 2;
- Threshold ratio for agricultural HRUs used for BMPs – 0.85.

#### Optimization procedure

The first iteration of GA runs was done for all water bodies that needed TN or/and TP load reductions to reach water protection targets represented by MAC. If the first run GA did not reach the required load reductions, it was repeated. This time, however, for a variable for which the required load reduction was not reached (TN or TP), a weight increase by 0.1 was performed. Rerunning GA for a particular sub-basin was completed if any of the following conditions were met: the required load reductions were reached, or maximum weight value was reached, or the threshold ratio for agricultural HRUs used for BMPs was crossed.

In the case of sub-basins with still unmet load reduction requirements, a second iteration was performed. Reductions unmet from the downstream sub-basin were transferred to the upstream sub-basins increasing their total load reduction requirements. Next, GA optimization was rerun in accordance with the aforementioned conditions. The procedure was complete if the required load reductions were achieved with additional measures in the upstream sub-basins, or if all possible upstream sub-basins were examined.

## Results

### BSAP and WFD – current state

Average nutrient loads calculated for the recent 5-year period (2016–2020) show that Lithuania is far above (exceeds) its BSAP NIC targets. For BAP, TN loads are 49630 t/y or 91.8% higher compared to NIC, while TP loads are 1163 t/y or 65.4% higher than NIC. It should be emphasized that nutrient loads generated from the territory of Lithuania are rising even further. Average loads for the previous 5-year period (2011–2015) were 39242 t/y or 20.9% lower for TN than in the current period. For TP, the loads were 821 t/y or 29.4% lower than in the recent 5-year period.

TN loading for GUR also exceeds NIC. Values for the recent 5-year period are 14898 t/y or 68.9% above NIC. TP loads for GUR, however, are 41.7% below NIC or 102 t/y. The loads of both nutrients for this basin also increased by 19.2% for TN and 10.8% for TP compared to the previous 5-year period. Average TN loads during the period 2011–2015 reached 12032 t/y TN and 91 t/y TP.

According to Aplinkos apsaugos agentūra (2021b), 41% of water bodies in the country are affected by agricultural activities sufficiently to be included in the risk water body category for the period 2016–2020. This is a significant increase from a 28% figure in the previous 5-year period (2011–2015). As previously mentioned, the intensification of agriculture and CC were the primary causes of these changes.

The numbers above clearly demonstrate that neither BSAP nor WFD water protection targets were achieved. Moreover, recent changes have further increased the gap between the actual state and the required targets. The root-causes for these negative trends were the intensification of agricultural activities and CC (Plunge and Gudas 2018). Our study, however, focuses solely on potential future impacts of pollution abatement measures and CC.

### Linkage between BSAP and WFD targets

TN and TP concentrations in water bodies were used as a reference point for the establishment of linkages between BSAP and WFD targets. WFD targets are rather straightforward and clear – these are “good” status concentrations, amounting to 3 mgN/l for TN and 0.14 mgP/l for TP in Lithuania. These targets do not change with CC. BSAP NIC TN and TP load targets were converted to concentrations using water flows obtained from modelling CC scenarios. Because water flows vary depending on meteorological data, the corresponding target concentrations to achieve NIC vary as well.

**Fig. 2:**
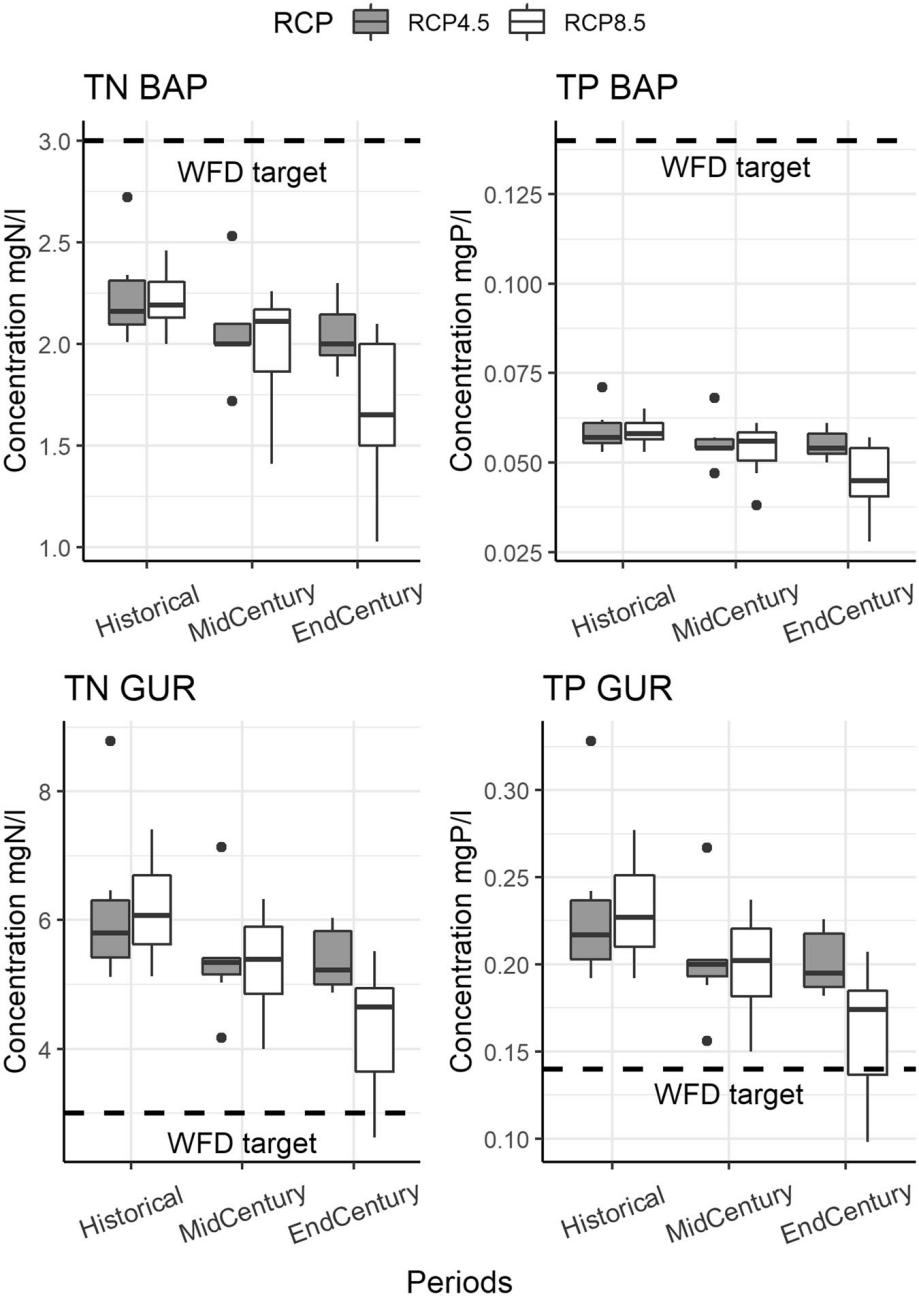
Target concentrations to reach BSAP NIC loads (box plots) and WFD target concentrations

Figure 2 presents results of the target concentrations to be achieved in the country’s water bodies. The box plots represent a range of concentrations needed to reach BSAP NIC targets (depending on flow conditions), while the WFD targets are presented as dashed lines. The results are presented for the BAP and GUR Baltic Sea sub-basins separately, because BSAP NIC targets and their ambition levels are different.

The results reveal the need for stricter concentrations in water bodies in order to reach NIC loads in the future due to changing climate. The greatest changes are expected with higher CC levels, and in a longer time frame. By the end-century in the worst-case CC scenario, the median target TN concentration should decrease by −24.7%, and TP by −22.4% for basins draining to the BAP Baltic Sea sub-basin. For GUR, the corresponding values are −23.4% for TN and −23.3% for TP. Another distinct feature is that target concentrations for WFD are much higher compared to what is needed for reaching BAP NIC loads. A particularly substantial gap occurs in the case of TP loads, where it is lower by −59.3% for median concentrations in the historical period. For TN, this gap is −28%. For the GUR, the situation is the opposite, because median target concentrations needed for GUR NIC achievement are less strict than the WFD targets. It should be emphasized though that in the case of the RCP8.5 scenario, for the end-century period, median target concentrations are becoming approximate to the WFD target ones. For the historical period, BSAP NIC load target median TN concentrations are 93.3% higher than the WFD target, and TP – 55% higher. The primary reason for the differences between target concentrations required for reaching BAP and GUR NIC targets are very different eutrophication indicator target levels set by HELCOM for the Baltic Sea sub-basins (HELCOM 2014).

### Required scale of the abatement program

The optimization results permitted the identification of the optimal strategy for each CC scenario and time horizon. The optimization methodology addressed the needs for TN and TP load reduction, yield loss prevention, and minimization of abatement program costs. Therefore the obtained results deliver a relatively balanced view of the necessary efforts to reach the country’s water protection goals. We acknowledge, however, that there are certainly multiple ways to reach the final goals. The results are highly dependent on how priorities are set by the decision makers. It is beyond our capabilities to obtain accurate information of this kind at a country level (it is a live process, multiple actors are involved, and much is to be decided in the future). Therefore, the results represent our identified best path forward.

**Fig. 3:**
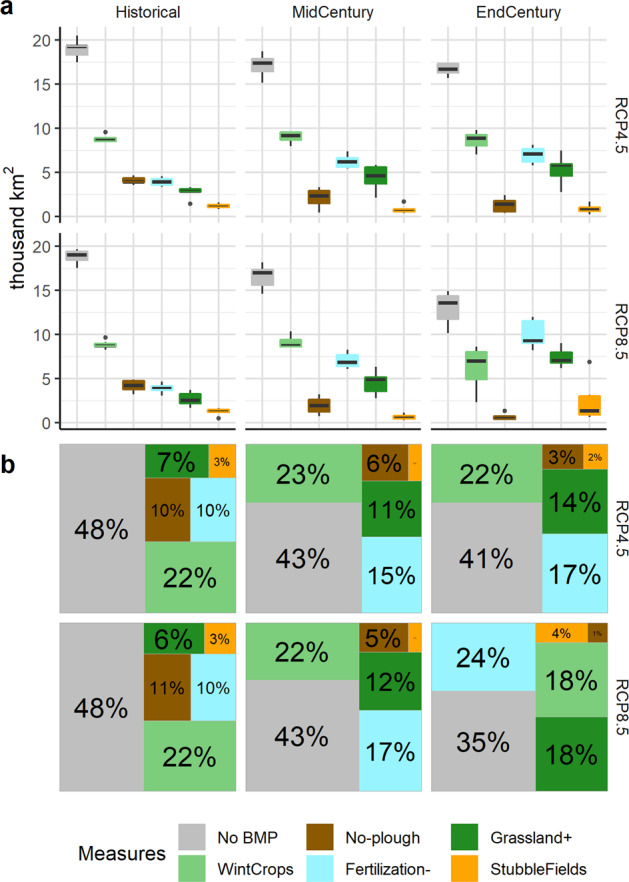
Distribution of BMPs on agricultural land required for reaching water protection goals. **a** Area distribution for all RCM models, **b** median distribution in percentages

Figure 3 presents the scale^5^ required for the agricultural diffuse water pollution program to reach water protection goals. It shows that in the historical period, roughly 52% of agricultural areas require some type of pollution abatement measures. CC for the intermediate scenario would require additional 7% of agricultural areas to be subject to BMPs until the end of the century (59% in total), while in the case of worst-case CC, an additional 13% of areas would require BMP application (65% in total).

It is important to emphasize that global warming is expected to alter the scale and priorities in the BMP selection. Only winter cover crops (except for RCP8.5 end-century period) would remain the primary measure to be applied on approximately one quarter of agricultural land in Lithuania. Stubble fields left through winter would also remain stable in their application area with CC, although it would manifest itself as the least important of 5 BMPs. The importance of the remaining measures, however, would significantly change, and this would involve opposite directions, depending on BMP. For instance, no-plough agriculture (the second most important measure of the historical period) would lose its significance by around 5-fold. On the other hand, CC would significantly increase the need for BMPs such as fertilization reduction and conversion to grasslands. In fact, fertilization reduction would become the main measure in the worst-case end-century CC scenario.

**Fig. 4:**
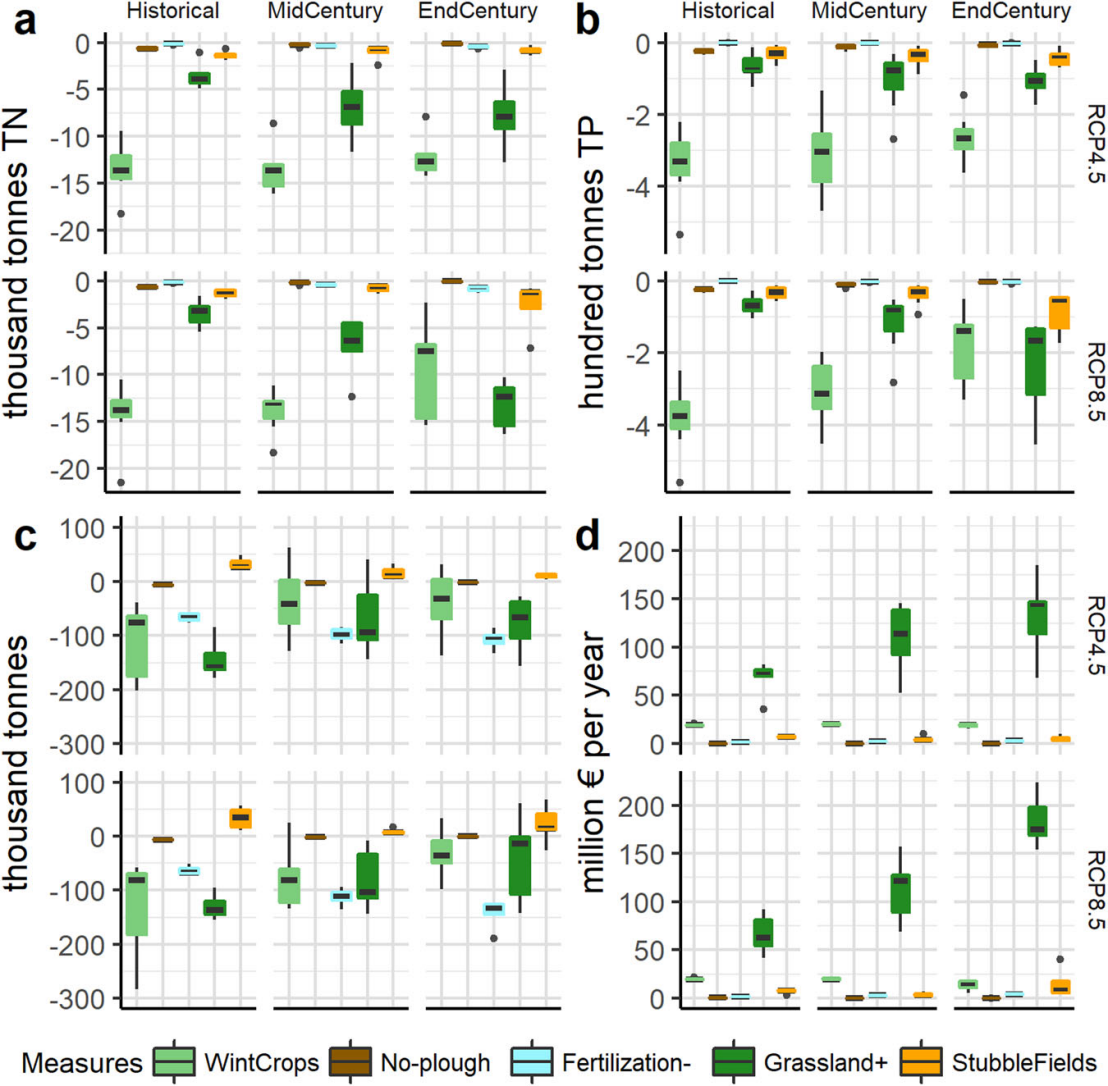
Cumulative (summed up for the entire territory of Lithuania) BMP optimization results for Lithuania for each BMP. **a** TN load reduction, **b** TP load reduction, **c** yield loss/gain, and **d** cost

The impacts of each measure on TN, TP load reduction, yield loss/gain, and costs are presented in Fig. 4. All the referenced variables were used for the optimization. The analysis of all 4 parts (A-D) reveals that overall, only two measures show possibilities for significant nutrient load reduction. These are winter cover crops and conversion to grasslands. Conversion to grasslands, however, comes with strings attached by inflating the costs of the possible pollution abatement program and yield loss for farmers. Winter cover crops, on the other hand, do not have such serious drawbacks. As a result, the use of winter cover crops seems to be crucial for reaching water protection goals and keeping abatement program costs low. It is particularly evident in the historical period. CC does somewhat lower the effectiveness of winter cover crops in reducing TN and TP loads, therefore prompting the application of additional measures in future time horizons.

It is worth mentioning that stubble fields might play an important part in reducing TP loads from agriculture. Firstly, the measure is relatively effective in reducing TP loads (only the winter crops and grasslands BMPs are more effective). Secondly, the measure has a positive impact on crop yields (as the only one), and would invoke comparatively low costs. Finally, this BMP appears to be relatively resilient to CC. Therefore, it could be a reliable and important tool for dealing with agricultural water pollution with TP.

On the other hand, no-plough and reduced fertilization BMPs seem to be of relatively low TN or TP load reduction potential (effectiveness). Notwithstanding this, the costs associated with these BMPs appear to be very low in comparison to those of other measures. Yet, future increases in yield losses could make the reduced fertilization measure less attractive.

**Fig. 5:**
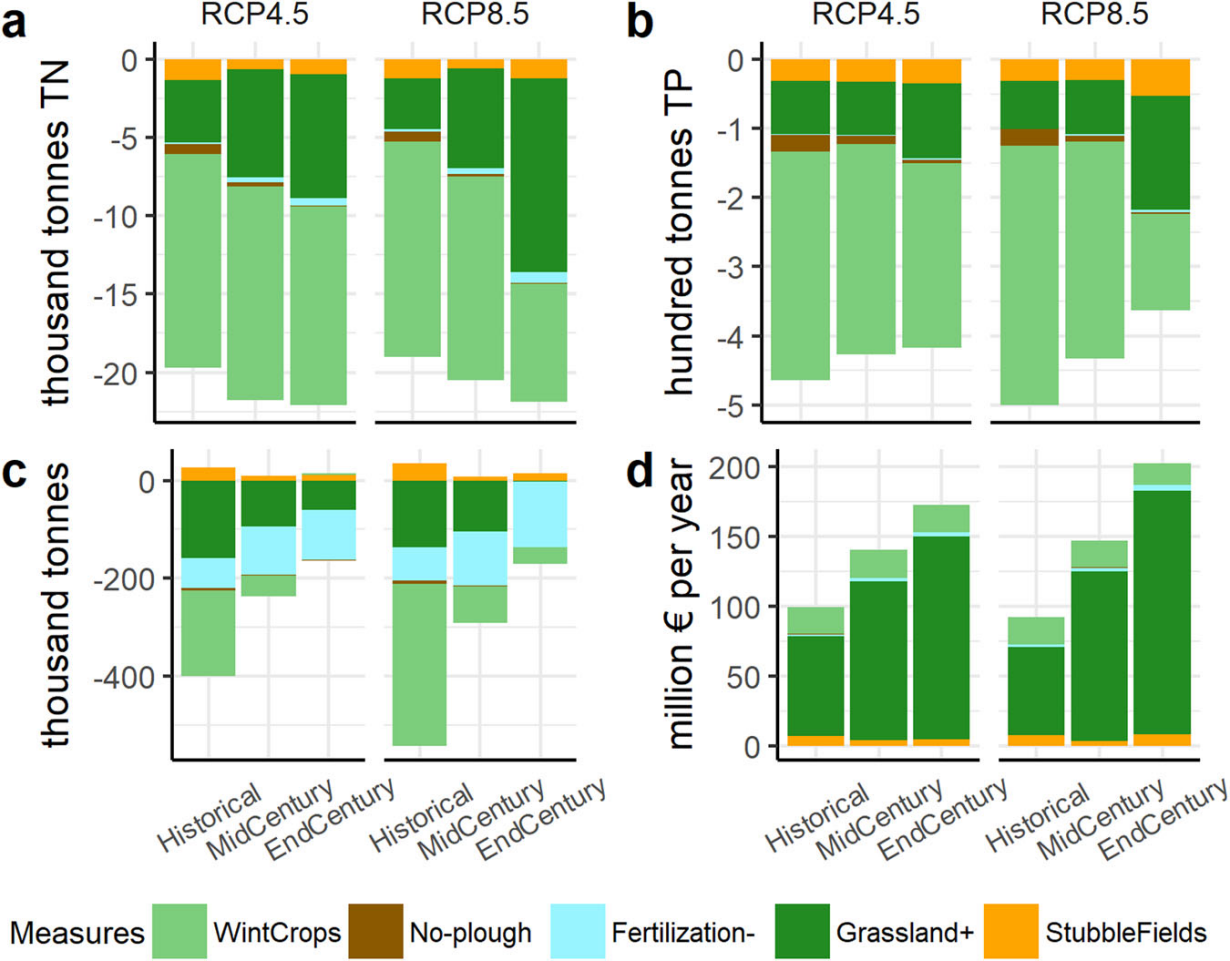
Median BMP optimization results for Lithuania for each BMP. **a** - TN load reduction, **b** TP load reduction, **c** yield loss/gain, and **d** cost

Cumulative median stacked results of BMPs in each CC scenario and time period are presented in Fig. 5. It demonstrates that the intensification of global warming would necessitate increased conversion of agricultural land into grasslands. Other measures are neither sufficient nor effective enough in changing climatic conditions. In the case of the RCP8.5 scenario, TN load reduction due to conversion of agricultural land into grasslands should be increased by 384% by the end of the century, while TN load reduction associated with winter cover crops would decrease by 45%. TP loads mirror the same trend as that observed with TN loads.

With regard to part C of the Fig. 4, a discrepancy is observed between the amount of yield loss for winter cover crops, particularly during the historical period. This variability results from the fact that yield optimization received the lowest weight of all variables used in the optimization. Therefore, the GA algorithm with each data set provided less consistent results. Notwithstanding this, the results show that yield loss from the application of BMPs will be a lesser problem in the future, with the exception of the reduced fertilization BMP. Nevertheless, an increase of the yield gap for fertilization could be primarily attributed to an increased application area of this BMP. Relative to area, yield loss would also be reduced. For example, for the worst-case CC scenario between the historical and end-century periods, yield loss per area due to the applied fertilization reduction BMP would decrease by 15%.

The cost results are quite clear – the grasslands would invoke the brunt of total costs associated with BMP implementation. The increased need for grasslands would significantly increase the costs of the program of agricultural diffuse water pollution abatement required to reach water protection goals. In both CC scenarios, those costs would substantially increase by end-century. For RCP8.5 CC, the costs would rise by 220% between the historical and end-century periods, while for RCP4.5 the increase would reach 173%. The difference between the worst-case and intermediate-scenario costs in the end-century period could be around 117% or 70 mln. euros in monetary terms.

It is however also important to mention that although an attempt was made to find optimal solutions to reduce pollution loads to the extent needed to reach WFD/BSAP targets, no possibilities were found by GA to fully accomplish this task for TN load reduction. In relation to the historical period, the remainder of the necessary reduction of 2075 tonnes of TN was not possible to achieve. The worst-case scenario additionally leaves 3595 tonnes of TN by the end of century on top of 5025 TN tonnes in the intermediate scenario (8621 TN tonnes in total), which would have to be reduced, yet no possibilities were found by GA. That would basically mean that in the worst-case scenario, with our optimized solution, TN targets for BAP would not be achieved, while the GUR and WFD targets would likely be complied with. Moreover, it should be emphasized that the presented costs represent optimal solutions with selected measures, applying BMPs in the most critical areas and at the exactly necessary amount to hit water protection goals. Yet, in real life, such conditions could not be expected. Furthermore, additional more effective BMPs, or the current BMPs at a broader scale, or combined use of them might be necessary to apply in order to fully achieve the required reductions. As a result, the real costs should be expected to be at a significantly higher level.

### Spatial distribution of selected BMPs and costs

**Fig. 6:**
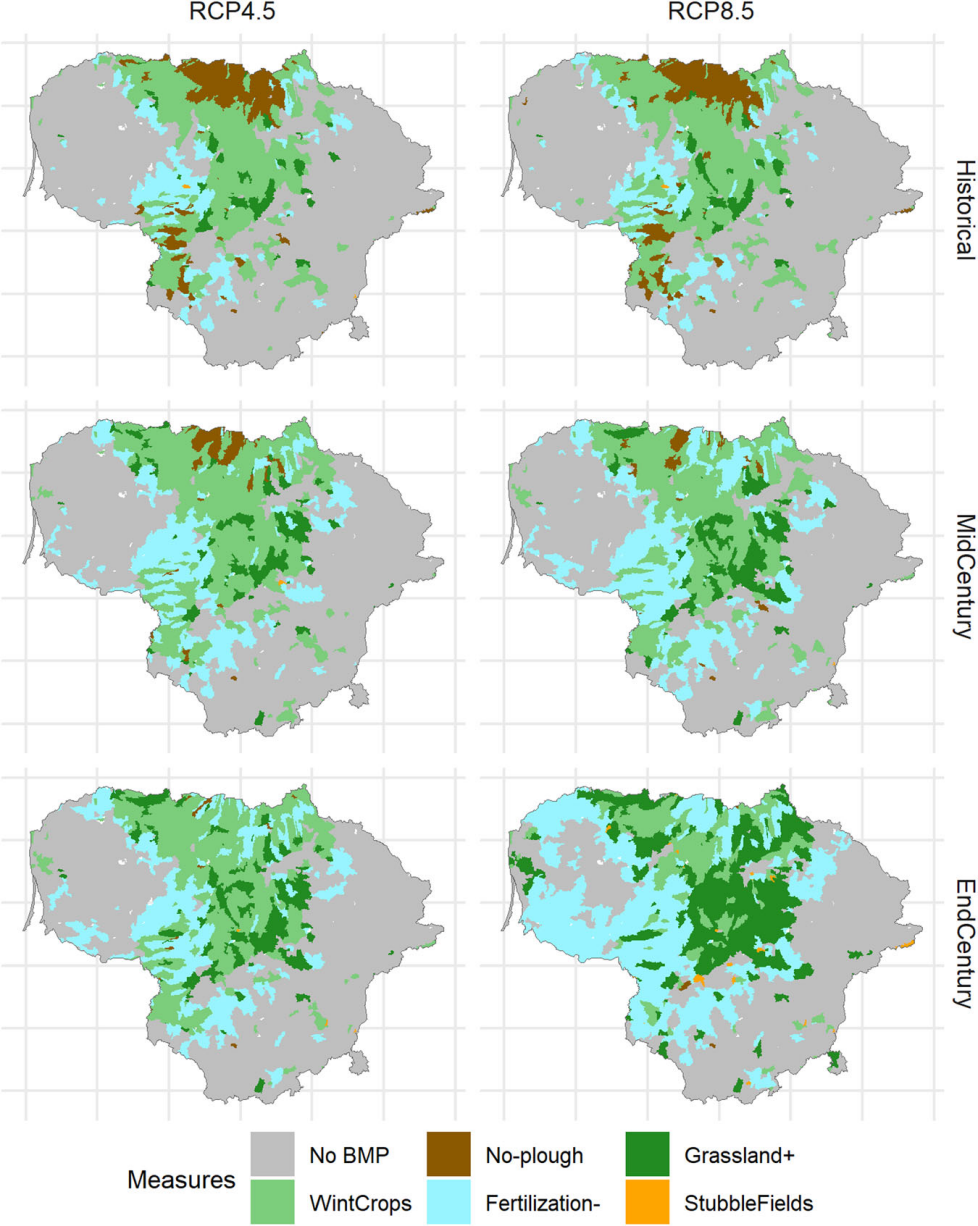
Distribution of dominant action as calculated from median optimization results per model sub-basin for CC scenarios and time scale horizons

Figure 6 presents the dominant (selected for the largest territory of agricultural land) option for each model sub-basin with each CC scenario and time horizon. It shows that the need to utilize agricultural pollution abatement measures is mainly confined to the central and northern parts of the country. This is no surprise – these areas are where most of Lithuania’s agricultural land is concentrated.

The results demonstrate that no-till agriculture should be the priority in the northernmost part of the country, as well as some sparse areas in its south-eastern part. This is, however, only true for the historical period. Due to a drop in surface runoff by mid-century, such areas will become a rarity, and will be non-existent by the end-century.

In the largest part of agricultural areas, priority should be given to winter cover crops. This BMP should be used in all agricultural areas where heavy TN load reduction is necessary. Only in the worst-case CC scenario for the end-century period, the extent of winter cover crop application should be matched by that of the grasslands BMP. Such change is due to the loss of cover crop effectiveness with changing climate, and the need to curb larger nutrient loads. As a result, with increasing intensity of global warming, more grasslands should be present in agriculture-dominated areas to tackle the nutrient load problem. This is especially evident in the central part of Lithuania.

As for the fertilization reduction, this BMP should be prioritized in areas where neither of the above-mentioned measures have already been prioritized. These are mainly territories adjacent to the most intensely used agricultural areas. In these areas, nutrient load reduction needs are much lower. A less effective but at the same time less costly fertilization BMP therefore seems to be a good fit there. As CC progresses, this BMP would be subject to increased prioritization and application in more and more areas as the effectiveness of some other BMPs (no-till, winter crops) declines. Moreover, the total area where reduced fertilization could be applied is significantly larger than that of other BMPs.

Stubble fields are not a priority measure in any of CC scenarios or time periods. Yet for some very specific small areas it might be an option, particularly for the end-century RCP8.5 CC scenario.

**Fig. 7:**
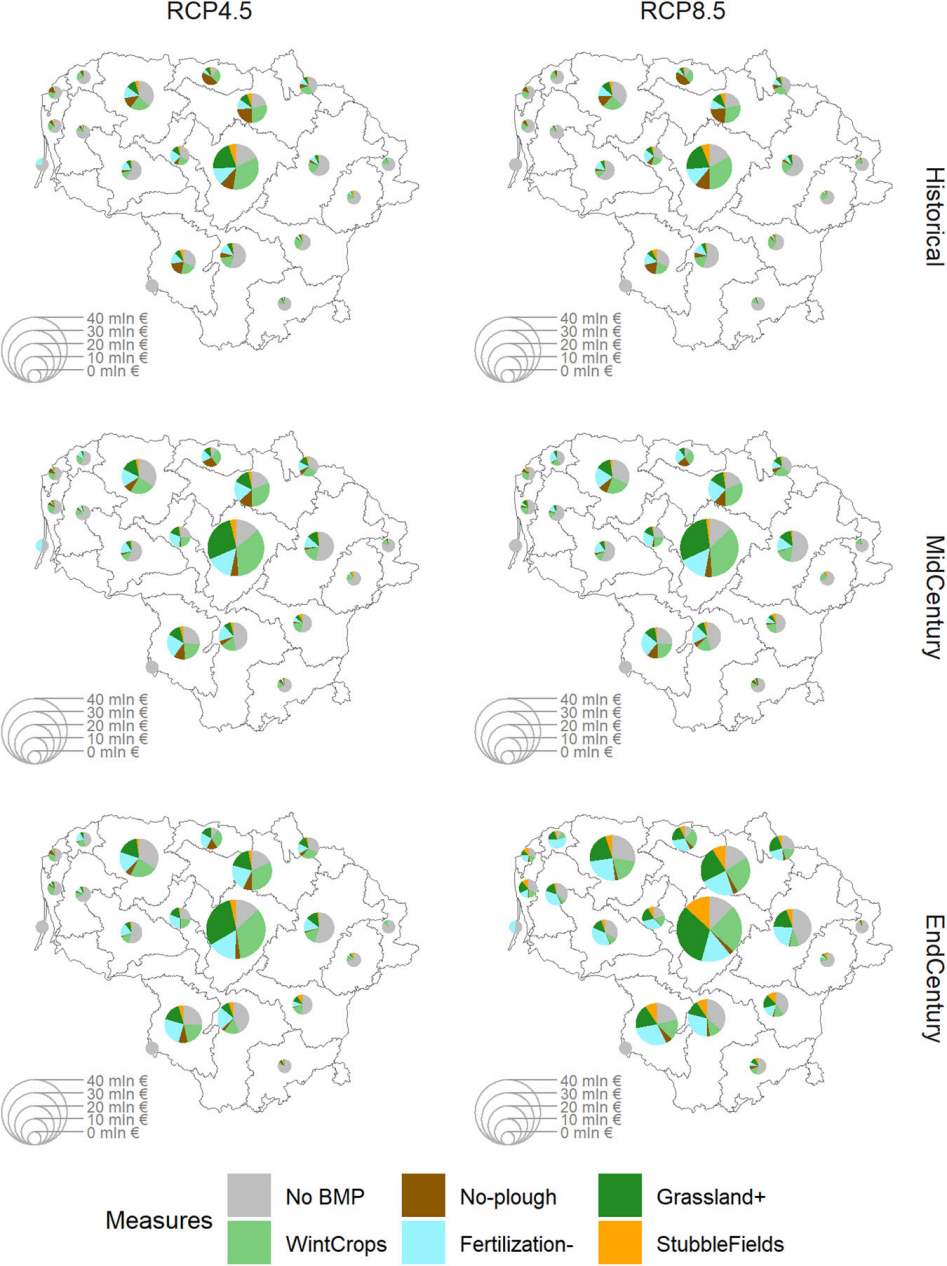
Composition of actions for agricultural areas (by share of BMP application area) aggregated per river sub-basin for CC scenarios and time scale horizons, and the corresponding abatement costs (in Euros)

Figure 7 presents the results for major river sub-basins in Lithuania as defined by Aplinkos apsaugos agentura^6^. Such a presentation provides a meaningful and basin-based (in line with water management principles) overview of major differences among separate parts of the country. The figure provides BMP distribution as well as overall agricultural diffuse pollution abatement costs per river sub-basin.

The results show that the northern and central sub-basins (dominated by agriculture) would require at least 3/4 of their area to be subject to some type of BMP. Winter cover crops and no-till BMPs should be the dominant choices closer to the historical period, while grasslands, reduced fertilization, and stubble fields left through winter BMPs should increase in importance with further global warming.

It is also important to emphasize that changing climate would leave less room for “business-as-usual” (no BMP) practices. This change would also increase the overall abatement costs per sub-basin needed to reach water protection goals. For instance, for the Nevezis sub-basin, the most problematic area in the middle of Lithuania, the costs of reaching water targets would increase by 42% for the RCP4.5 scenario and by 61% for the RCP8.5 scenario between the historical and end-century periods. In the case of the Musa River, the second most problematic river sub-basin in Lithuania just north of the Nevezis sub-basin, the cost increases reach 63% for the RCP4.5 scenario and 115% for RCP8.5, respectively.

Notice that not less than 15% of each sub-basin area is devoted to the no BMP option, even in the case of the most problematic river sub-basins. Such a situation results from setting the threshold ratio parameter for agricultural HRUs used for BMPs at 0.85 in GA optimization. This parameter allowed for speeding up the optimization. Moreover, it also permitted representing a more realistic situation by keeping space for “business-as-usual” practices, because a 100% application rate of BMPs on agricultural land is not realistic due to multiple constraints connected with agricultural practices, or even psychological factors.

## Discussion

### Misaligned and changing water protection targets

One result of the study was the finding that accounting for CC could also raise questions about water protection targets themselves. BSAP targets are expressed in NIC TN and TP loads to the Baltic Sea (HELCOM 2021). Therefore they have to be transposed into units comparable to inland WFD water targets, defined in concentrations. After BSAP target conversion into TN and TP concentrations, it became clear that the target concentrations (required to reach BSAP targets) are expected to decrease (become more strict) due to CC induced increased water flows.

According to Poikane et al. (2019), WFD TN and TP target concentrations for rivers set by EU countries were in a range of 0.25–35 mgN/l with a median at 2.5 mgN/l for nitrogen, whereas for phosphorus the range was 0.008–0.66 mgP/l with a median at 0.1 mgP/l. Although the concentrations required to reach Lithuania’s BSAP targets are within those ranges, their considerable misalignment is observed with Lithuanian WFD goals. It is particularly evident in the comparison of Lithuanian WFD goals to BSAP target concentrations for the BAP Baltic Sea basin area. TN concentrations required to reach BSAP goals are in a range of 1.65–2.19 mgN/l, whereas WFD target concentration is set at 3 mgN/l. The same problem occurs for TP concentrations were the necessary level for BSAP is in a range of 0.045–0.058 mgP/l, while the WFD target is set at 0.14 mgP/l.

An additional discrepancy stems from different levels of ambition set by HELCOM in defining “good” status indicator values for BAP and GUR. According to HELCOM (2014), for the main part of the BAP basin (Eastern Gotland basin), “good” status winter dissolved inorganic nitrogen is set at 2.6 mgN/l, while in GUR basin it is 5.2 mgN/l. The same pattern holds for winter dissolved inorganic phosphorus “good” status concentrations, reaching 0.29 mgP/l for BAP and 0.41 mgP/l for GUR. Other criteria are set with the same bias. Therefore, for GUR, inland TN and TP target concentrations are in a range of 4.65–6.07 mgN/l and 0.174–0.227 mgP/l, respectively, which is far from the ranges for BAP basin target concentrations.

The aforementioned discrepancies also mean that in the Nemunas and Venta River basin districts (RBDs), Lithuania should do more than it is required under the WFD targets in order to reach stricter BSAP goals for the BAP Baltic Sea basin area. Contrary to the Nemunas and Venta RBDs, in the remaining RBDs, reaching WFD targets would be more than sufficient to achieve less stringent BSAP objectives for the GUR Baltic Sea basin area.

### Increasing costs of reaching water targets under CC

The results of this study suggest that reaching water protection goals while accounting only for current climatic conditions would be insufficient for Lithuania. Even after the elaboration of a CC-optimized pollution reduction program in this study, it appeared that in the worst-case end-century CC scenario, roughly 30% of TN reduction required to meet BSAP BAP targets would be left unfulfilled. This means additional measures would be necessary to achieve the goals, including expanding the extent of the application of the same BMPs, or utilization of other possibly more effective, but also more costly BMPs, such as nutrient retention measures, etc. As a result, the growth trajectory of the program implementation costs would become even steeper.

The CC-related cost inflation problem has also been demonstrated in studies from other countries. In a relevant study from a small US watershed (298 km^2^), Renkenberger et al. (2017) concluded that BMPs designed to reach water protection goals under current conditions will become insufficient with CC. The suggested recommendation was that whenever possible, BMP design and implementation programs should focus on the expected climatic conditions rather than on the current ones. Such an approach would help avoid costly redesigns of the water protection program. Another US study on a small catchment (7.3 m^2^) by Bosch et al. (2018) concluded that CC could affect the relative profitability of crops. It would also increase pollution loading from crops, and change costs and effectiveness of BMPs, all of which would change the ability to meet water quality goals. The study by Qiu et al. (2020) focused on a larger watershed (14,924 km^2^) in China. The study results demonstrated that the efficiency of BMPs under future climatic conditions would decrease, whereas TN, TP, and sediment loads to the streams would increase.

Shared Socio-Economic Pathways besides CC scenarios were not considered in the study. However, taking into account possible changes in land use, agricultural practices, energy demand, population growth, food production, etc., are important for exploring the long-term consequences of CC and suitable options for the response (Kriegler et al. 2012). For instance, CC impacts on global agricultural production compounded with a worldwide increase of meat and dairy consumption could put growing demand on the Baltic region for the expansion of agricultural production. It would result in increased livestock production and more agricultural land use, focus on high productivity in large-scale farms, reduced N and P efficiencies for increased exports of agricultural products, which would put additional pressures on the water environment (Zandersen et al. 2019). According to Wulff et al. (2014) increased nutrient loads should be expected from increased intensity of agriculture in BSR intransient countries (such as Poland, the Baltic States and Russia). Trends in that direction confirmed by monitoring results in Lithuania (Plunge and Gudas 2018).

Therefore, such tendencies would certainly require additional measures in protecting water resources. Decision makers and water managers have to be prepared to consider impacts of the changing environment. Universities should be also better adapted to include uncertainties and unpredictability of climate change as well as socio-economic changes in training the next generation of watershed decision-makers (Perry and Thompson 2019). A special focus should be on climate resilient and adaptable measures such as nature-based solutions, which by enhancing natural capital could provide cost-effective, resource-efficient and competitive circular economies with environmental, social and economic benefits coming from it (UN Water 2018).

### BMP targeting

Our results were based on the application of an optimization algorithm that selected optimal locations and the most efficient measures to reach water protection goals while keeping the costs low. Such conditions can be hardly expected in real life. Targeted assignment and implementation of measures at a local scale would require adaptation with appropriate legal and administrative tools, precise information, support from the farmer community, and other conditions. Our outcomes should therefore be treated only as best-case scenario results. Nevertheless, it is impossible to overestimate the importance of the application of the targeted approach (in terms of measures and their locations) for reducing the overall costs of meeting water protection goals.

The identified highly vulnerable areas or critical source areas should be targeted for the application of effective BMPs (Giri et al. 2020; Xu et al., 2019). It is necessary to develop cost-effective strategies to meet water protection goals under CC in order to mitigate the CC consequences in the watershed. According to Piniewski et al. (2021), as current water protection programs are yet to fully embrace the targeting approach, there is a huge untapped potential for reducing pollution removal costs. One example is provided in the study by Xu et al. (2019), demonstrating how the targeting costs could be halved only by using targeting. Findings of Wagena and Easton (2018) showed that targeting is an important tool for water managers to provide water quality results while avoiding rising costs due to the need to increase BMPs adoption.

### Sufficiency of existing and planned measures

It is important to examine how our results compare to the existing water pollution reduction programs in Lithuania. According to ZUIKVC (2022), in the year 2021, the application of BMPs (also used in our study) in the country was as follows: no-till agriculture was applied on 7.84% of arable land, stubble fields – 1.31%, winter cover crops – 1.15%, conversion to grasslands – 0.23%, whereas fertilization reduction was applied on 0%. The application scale is certainly far from adequate to achieve any noticeable improvement in water ecosystems. If no-till agriculture (required coverage in our results is 10-11% of arable land for the historical period) and stubble fields (required coverage – 3%) are increased by certain percentages, other measures (required coverage in our results for winter cover crops – 22%, reduction of fertilization – 10%, conversion to grasslands 6-7% of coverage requirement) should be scaled up by orders of magnitude.

Some improvements could however be anticipated from the planned actions. For instance, in its National Energy and Climate Action Plan, the Ministry of Environment of the Republic of Lithuania (2020) sets the task to increase no-till areas to 20.97% of arable land by 2030. Balanced use of mineral fertilizers is also planned, which should reduce the overall use of mineral N fertilizers by 15%. Meanwhile, the Ministry of Agriculture of the Republic of Lithuania (2022) plans to increase the coverage of land by winter cover crops to 2.42% of arable land by 2027.

It is not clear if such planned actions would materialize, considering that similar provisions have also been included in official planning documents before. The progressively increasing use of mineral fertilizers, and the intensification of agricultural methods in Lithuania have significantly increased the levels of nutrient loads to water bodies (Plunge and Gudas 2018). This, in addition to CC impacts, makes it even more difficult to meet water protection goals. According to Aplinkos apsaugos agentūra (2021b), the overall result was an increase in the number of water bodies at risk due to agricultural pressures from 28% during the period 2010–2015 to 41% of all water bodies in the years 2016–2020. It should be pointed out that our modelled BMP of reduced fertilization translates to 10% reduction from optimal plant fertilization. There is plenty of evidence that in intensive agriculture areas (central and northern parts of Lithuania), the application of N fertilization sometimes reaches 600 kg N/ha/year, which is far from optimal (CATCH POLLUTION 2019a).

### Cost-effective strategies

The analysis of the existing or planned measures in the context of our results reveals that winter cover crops is probably the most underutilized measure, and therefore it could be considered as the lowest-hanging fruit. According to the CATCH POLLUTION (2019b) report, even if costs for farmers are up to 5 times higher compared to the reduced amount of used fertilizers, with consideration of other benefits (reduction of nutrient leaching, nutrient transfer to the next crop, increased soil organic carbon content, reduced soil erosion, reduction of GHG emissions, control of weeds), the potential overall gains outweigh the incurred costs (as much as 100 times). Moreover, CATCH POLLUTION (2019c) estimates that without changing the crop structure in the country, winter catch crops could be applied to at least 20% of arable land. Therefore, in all CC scenarios, increased investment into the implementation of this BMP is the most straightforward route towards reaching water protection goals.

Another pathway, potentially crucial, especially with consideration of CC impacts, is controlling and limiting fertilization, particularly with mineral fertilizers. The cost of this measure for farmers recalculated from yield loss might be substantial only if optimal fertilization level is assumed. In the case of fertilizer overuse, however, increased operation costs as well as income loss for farms from potential yield loss (Albornoz 2016) do not make much sense, even without taking environmental costs into account. Therefore, controlling and limiting fertilization is one of the most critical and climate resilient tools (suggested by our results) for the protection of water ecosystems.

Depending on the CC scenario, arable land conversion to grasslands could become increasingly important, particularly in the long term and the worst-case CC scenario. In such cases, much larger nutrient load reductions would be necessary, necessitating more effective, although most likely more expensive solutions. Nevertheless, in terms of environmental effects, grasslands would have a positive impact not only on the protection of water bodies, but also on carbon sequestration (Lorenz and Lal 2018), which is becoming increasingly important worldwide in order to reach UN (2015) agreement goals, and to avoid the worst-case CC scenarios. Our results generally suggest that under more pessimistic CC scenarios, less arable land could actually be used for crop production if water protection targets are to be met.

On the one hand, a significant reduction of agricultural land use appears possible for the country as only around 2.5% of its gross domestic product comes from agriculture, fisheries and forestry combined (Statistics Lithuania 2019). Moreover, Lithuania’s agriculture is heavily focused on grain production, which takes up around 77% of all arable land (Statistics Lithuania 2021). According to FAO (2020) around two-thirds of the country’s grain production is exported as raw material, which is heavily supported by EU Common agricultural policy subsidies. For instance, in the 2014–2018 period on average 71% of farm income was coming from EU subsidies (EC 2020), whereas less than 6% of the country’s workforce was employed in the agriculture, forestry and fisheries sectors (Statistics Lithuania 2022). Furthermore, the involvement of the workforce in those sectors is steadily decreasing (i.e. in 1995 was 19%). Thus, socio-economic factors show that changing the agricultural model to less intensive could be possible. On the other hand, food security concerns in the changing climate and geopolitical context as well as the political sensitivity of agriculture sector-related issues might pose a serious obstacle for such kind of a transition.

## Conclusions

Many countries are facing a common challenge of simultaneous reaching of water protection goals in inland and the marine waters as agriculture remains the major pollution source. The required pollution reductions and the related cost implications are usually substantial, necessitating optimization tools for reaching water objectives with the lowest costs possible. Climate change brings in additional challenges in the pursuit of water protection goals, in many cases potentially exacerbating the problem. In this context, this study attempted to find out what it would take for Lithuania to reach its inland and marine water-related targets in terms of required agricultural diffuse pollution reductions and costs by using the targeted approach, optimizing BMP selection spatially based on BMP effectiveness, costs, and yield loss.

The study revealed the already existing discrepancy in the hierarchy of goals before even starting optimization of a BMP set for Lithuania. Recalculated HELCOM BSAP nutrient loads to concentration targets for rivers flowing to BAP are much more strict than for those flowing to GUR. The same river concentration targets for BAP are much more strict than WFD target concentrations, while for GUR inflowing rivers the situation is the opposite. This implies the need for Lithuania to develop pollution reduction measures for BAP more ambitious than those required by WFD, while for GUR rivers, less ambitious WFD-compliant measures will be sufficient. CC will tend to lower BSAP-related target concentrations (by approximately 23%), making the goal misalignment for BAP even larger.

The results suggest that in order to be close to reaching water objectives, BMPs should be applied in at least 52% of agricultural areas. This number should increase to 65% for the worst-case (RCP8.5) end-century CC scenario, which in turn would mean less arable land available for crops in the future if water targets are met. The high costs of reaching water targets would tend to rise even more with CC – by 173% for RCP4.5, and by 220% for the RCP8.5 CC scenario, approaching a value of 200 million euros/year. In such a context, the targeted BMP optimization approach is essential for significant lowering of the costs, because without optimization the costs will be much higher.

CC would change the significance of some BMPs, while the role of other BMPs would remain unaltered. Winter cover crops and stubble fields left through winter are expected to remain among the most and least important measures, respectively, throughout all CC scenarios. However, no-plough agriculture (second most important BMP in the historical period) is expected to become unimportant, while land conversion to grasslands, and particularly reduced fertilization measures, would be listed among the most important ones.

Winter cover crops and reduced fertilization show the best effectiveness and cost balance, and will therefore be essential in pursuing water protection targets. Those measures would be the most important to be broadly applied in the future, especially that those BMPs are currently utilized at a scale significantly lower than necessary (more than 5 times less). Moreover, those measures are highly cost-effective, and provide other benefits to farmers (weed, erosion control, nutrient savings, etc.). Conversion to grasslands is among the most effective measures, although due to its high costs and yield losses it is considered a last resort measure. The severity of the worst-case (RCP8.5) end-century CC scenario impacts, however, would require substantial expansion of this last resort measure application (TN load reduction by this BMP would increase by 384%), severely inflating the costs of implementation of the water pollution reduction program.

In spatial terms, winter cover crops and conversion to grasslands should be prioritized in the lowlands of northern and central Lithuania, where reductions are needed. Reduced fertilization in the historical period would act as a supplementary measure adjacent to intensive agriculture regions, whereas due to its relatively low costs and sufficient effectiveness with CC, its application extent in agricultural areas should substantially increase.

CC would make the scale of the optimized program of pollution abatement measures insufficient to reach BSAP BAP targets for TN. In the worst-case (RCP8.5) end-century CC scenario, approximately 30% of needed reductions would not be attained. In order to reach those targets, a broader application of BMPs, or additional more effective although potentially more costly types of BMPs (like wetlands, sedimentation ponds, afforestation, etc.) should be considered, driving the program costs even further.

## Footnotes

1 The report is available at: https://vanduo.old.gamta.lt/files/3%20priedas_SWAT%20modelis_20150817_SD.doc

2 Data available at: https://www.euro-cordex.net/

3 Degree of error will be present due to the assumption all water bodies have the same concentration.

4 This is the smallest spatial unit in the SWAT model, identified by unique land use, soil, and slope characteristics in a model sub-basin.

5 The combined total area for measures represented in the figure is around 7% larger than country area due to inclusion of transboundary areas drained by small rivers across the border.

6 Source map and description available at: https://vanduo.old.gamta.lt/cms/index?rubricId=b649c5d3-8be2-4af4-a186-c0aed3a4555f
